# Endoscopic endonasal pituitary adenomas surgery: the surgical experience of 178 consecutive patients and learning curve of two neurosurgeons

**DOI:** 10.1186/s12883-016-0767-0

**Published:** 2016-11-30

**Authors:** Xuefei Shou, Ming Shen, Qilin Zhang, Yichao Zhang, Wenqiang He, Zengyi Ma, Yao Zhao, Shiqi Li, Yongfei Wang

**Affiliations:** Department of Neurosurgery, Shanghai Pituitary Tumor Center, Hua Shan Hospital, Shanghai Medical College, FuDan University, Shanghai, China

**Keywords:** Endoscope, Pituitary adenoma, Endonasal approach

## Abstract

**Background:**

We aim to study surgical technique and analyze the related factors affecting tumor total removal and postoperative endocrinological remission for endoscopic endonasal pituitary adenomas surgery.

**Methods:**

We retrospectively analyzed 178 endoscopic endonasal pituitary adenomas surgery from March 2011 to May 2014. Endonasal approach included the routine transnasal-sphenoidal approach, transnasal- maxillary sinus approach in four cases and transnasal-clivus approach in one case.

**Results:**

According to postoperative imaging data and endocrine examination results, total removal was achieved in 129 patients (72.5%), and endocrinological remission was achieved in 38 patients with functional adenomas (44.1%). Statistical analysis of the clinical data showed that total removal rate was much closely related to tumor volume (*P* = 0.006), and tumor invasiveness (*P* < 0.001).

**Conclusions:**

In this study, we found tumor sizes and invasion of cavernous sinus were related to total removal rate and endocrinological remission rate; the direction and degree of tumor invasion, and the surgeon’s experience were the key influence factors of the endocrinological remission rate for invasive functional pituitary adenomas.

## Background

Trans-sphenoidal surgery for pituitary adenomas was firstly invented by Herman Schloffer more than 100 years ago [1]. It was then developed from trans-sphenoidal surgery by microscope into endoscope-assisted microscope, and nowadays has progressed to pure endoscopic endonasal surgery [2, 3]. The purpose of this article is to introduce the experience of endoscopic endonasal surgery for pituitary adenomas by a group of two neurosurgeons in recent 3 years, and how we improve our surgical techniche through practice, at mean time to discuss the pros and cons of the endoscope in this approach.

## Methods

1. Clinical materials: from March 2011 to May 2014, 178 endoscopic endonasal operations were performed on patients with pituitary adenomas. Among these, 78 cases were male patients and 100 cases were female patients, ranged from 14 to 74 years old (mean 46.1 years). The main clinical features were: 65 cases with impairment of version and visual field, 6 cases with ocular motility disorders, 28 cases of menstrual disorder and secondary amenorrhea(in prolactinomas, some GH-secreting adenomas and plurihormonal adenomas), six cases with galactorrhea, 25 cases with male sexual dysfunction, 49 cases with acromegaly, 4 cases with central obesity and hirsutism, one case with developmental retardation, and 19 patients underwent a reoperation for tumor recurrence.
2. Radiographic examination: Preoperative sellar coronary CT and MR examinations were performed in all patients. Modified Hardy classification was utilized according to the size of tumor. 108 cases (60.7%) were invasive adenomas and 70 cases (39.3%) were non-invasive adenomas (Table 1). The direction of tumor invasion: prolactinomas were prone to invade laterally (72%,13/18);GH-secreting adenomas were prone to invade inferiorly (45%,13/29); Non-functional adenomas can invade in all directions.

**Table 1:** General material

	PRL	GH	ACTH	Pluri + TSH	Nonfunctional	Total
Number	32	43	4	7	92	178
Gender						
Male	13	20	0	3	42	78 (43.8%)
Female	19	23	4	4	50	100 (56.2%)
Invasiveness						
Non	14	14	2	2	38	70 (39.3%)
Invasive*	18	29	2	5	54	108 (60.7%)
Hardy Grade						
I	6	6	2	0	3	17 (9.5%)
II	12	21	1	4	20	58 (32.6%)
III	11	14	0	2	45	72 (40.4%)
IV	3	2	1	1	24	31 (17.4%)
Removal rate						
Total	25	31	3	4	66	129 (72.5%)
Near total	6	7	1	3	16	33 (18.5%)
Subtotal	1	5	0	0	10	16 (9.0%)

Invasive*: pituitary adenomas with suprasellar or parasellar or infrasellar extension
3. Endocrinological Evaluation: All patients underwent complete preoperative endocrinological evaluation: four cases had abnormal elevation of plasma cortisol and ACTH. three cases had abnormal elevation of thyroid hormones, 49 cases had abnormal elevation of GH and IGF-1, 16 cases had slight elevation of PRL(<100 ng/mL), 30 cases had significant elevation of PRL(>100 ng/mL).
4. Surgical methods: The instruments including 0°, 30°, wide-angle lens, 4-mm diameter rigid endoscopes (Karl Storz, Germany), endoscopic surgical instruments (Karl Storz, Germany), IPC power system and endoscope irrigation system(Medtronic, USA). And Selectable Depth Doppler monitor system (VTI, USA) was generally used.
  All patients were treated by the same group of two neurosurgeons (Dr. Yongfei Wang and Dr. Xuefei Shou), without the help of ENT colleagues. Surgical procedure: the right nostril was usually chosen as the operation channel, unless the tumor obviously invaded the right cavernous sinus or the right nostril was too narrow to proceed. In the case that tumor was large or bilateral cavernous sinuses were invaded, both nostrils were used, and one or bilateral middle turbinates were removed to gain more operative space. After the sphenoid sinus was opened, sellar dura was fully exposed according to the size and location of the tumor (the routine transnasal-sphenoidal approach). To expose the tumor, if the posterior ethmoid sinus or anterior skull base was invaded, the ipsilateral middle turbinate would be removed (transnasal- ethmoid sinus approach); if maxillary sinus or pteryogalatine fossae was invaded, the ipsilateral middle turbinate and uncinate process would be removed (transnasal-maxillary sinus approach); if lower clivus or foramen magnum was invaded, the middle and lower clivus would be removed (transnasal-clivus approach). All the microadenomas underwent extracapsular dissection while macroadenomas were resected piecemeally (intrasellar firstly, then suprasellar, finally, explore the bilateral medial wall of cavernous sinus and remove the residual tumor invading into the cavernous sinus if possible). Repair the sellar diaphragma intentionally if any defect was found.
5. Postoperative nosocomial evaluation and outpatient follow-up: all the patients enrolled underwent a total endocrine evaluation on the first postoperative day and MRI scan within 3 days after the surgery. The endocrine function was assessed in the 1 month, 3 months, 6 months and each year after discharge. MRI scan was advised each time since the second follow-up (the third postoperative month). The criteria for endocrinological remission of GH-secreting adenoma were: OGTT nadir GH level < 0.4 ug/L or random GH level < 1 ug/L and IGF-1 level was at normal range according to the age and sex simultaneously. The normal range for PRL was <22.80 ug/L for male, and <30.74 ug/L for female.
6. Learning curve: we separate the 178 cases into stage one (89 cases) and stage two (89 cases), which was treated 18 months formerly and 20 months later, respectively.
7. Statistical Evaluation: chi-square test, Fisher’s exact test and Logistic regression analysis were used. A *p* value < 0.05 was considered significant. Statistical analyses were performed using SPSS software (version 17.0 for Macintosh).

## Results

This study included nonfunctioning adenomas in 92 cases, prolactinomas in 32 cases, GH-secreting adenomas in 43 cases, plurihormonal adenomas in seven cases and ACTH-secreting adenomas in four cases. All the diagnoses were based on pathological, immunohistochemical and clinical evaluation.

Among the 178 cases, besides the routine transnasal-sphenoidal approach, the transnasal- maxillary sinus approach was in four cases, and the transnasal-clivus approach was in one case. According to postoperative imaging data, total removal was achieved in 129 patients, near total removal was in 33 and subtotal removal was in 16. Endocrinological remission was achieved in 38 patients with functional adenomas (44.1%) (Table 1).

**Fig. 1:**
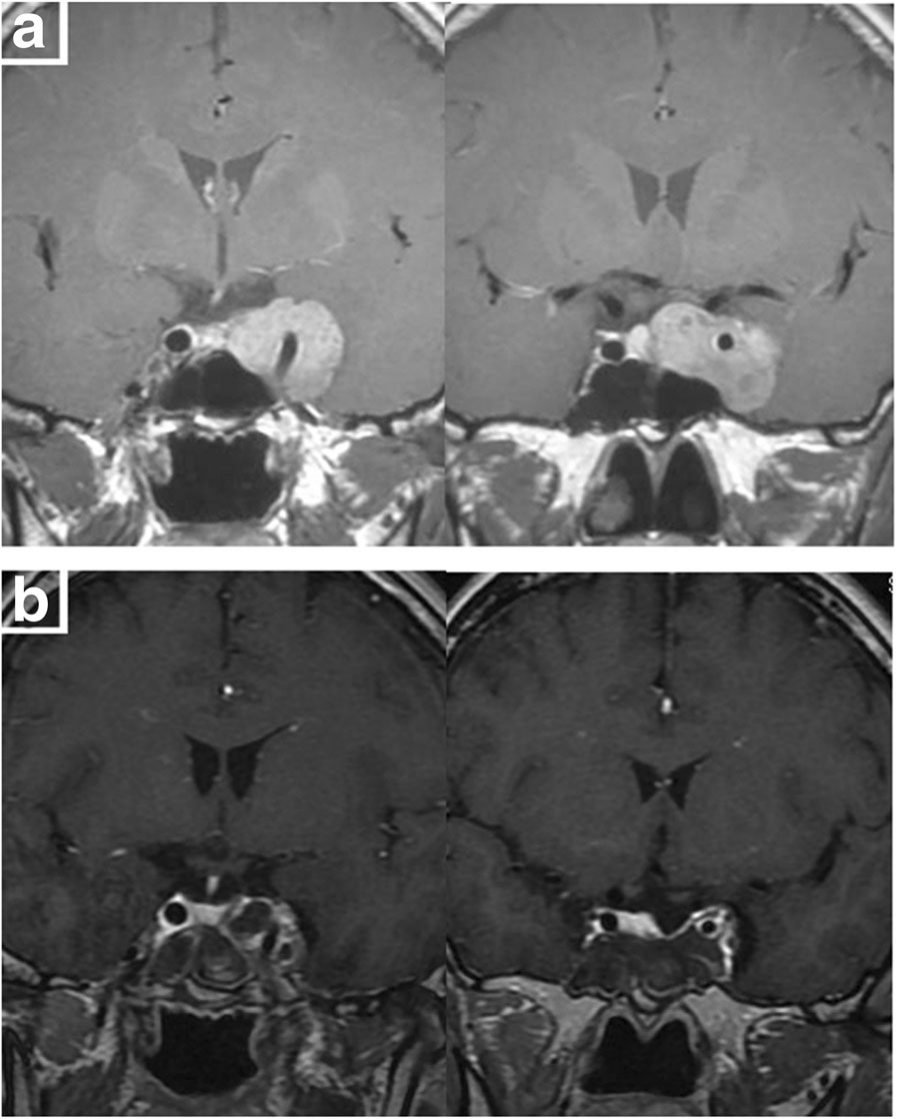
**a** Preoperative MRI shows left cavernous sinus invasion, encasing left internal carotid artery. **b** Postoperative MRI shows the tumor invading into cavernous sinus was mostly excised

**Fig. 2:**
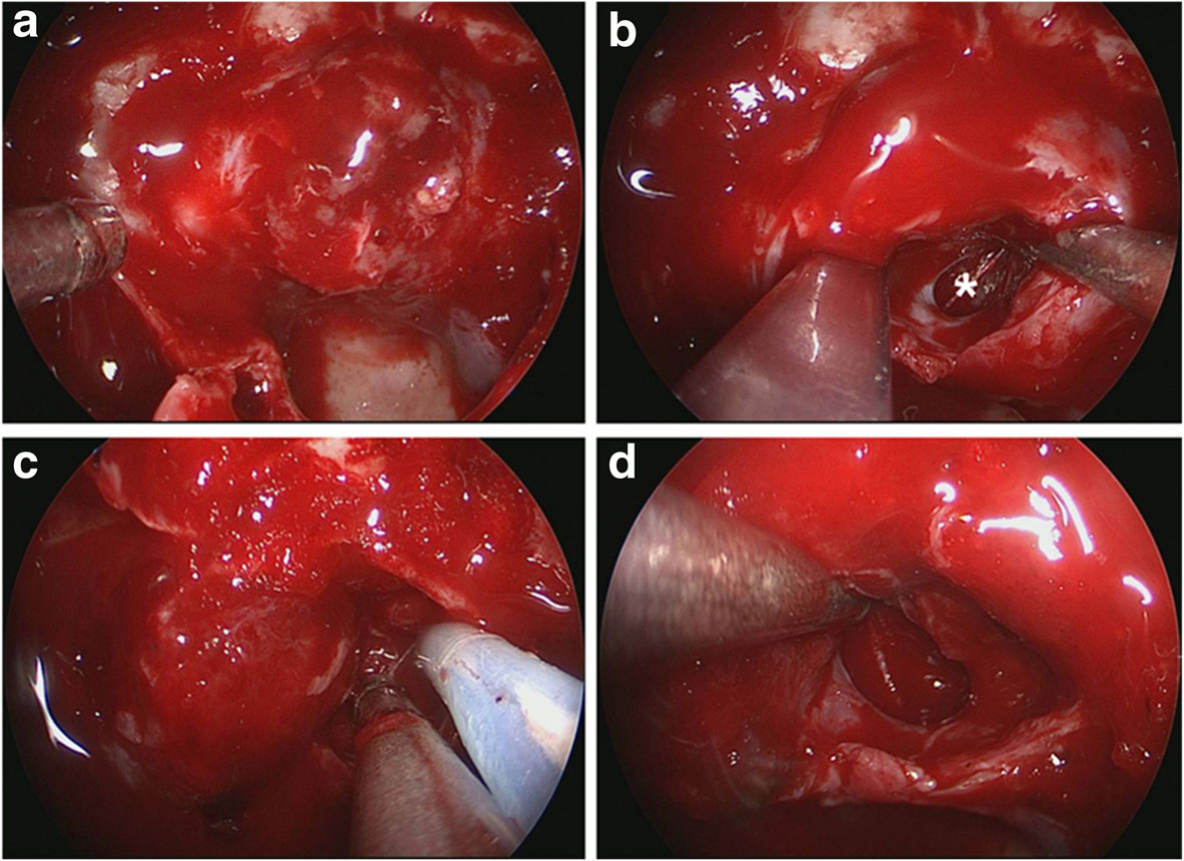
**a** The sellar floor was open to expose the tumor. **b** The tumor invading left cavernous sinus was removed. *marks the defect of the medial wall of cavernous sinus. **c** Cavernous ICA is localized by intraoperative Doppler ultrasound. **d** No residue tumor was observed under endoscopy

**Fig. 3:**
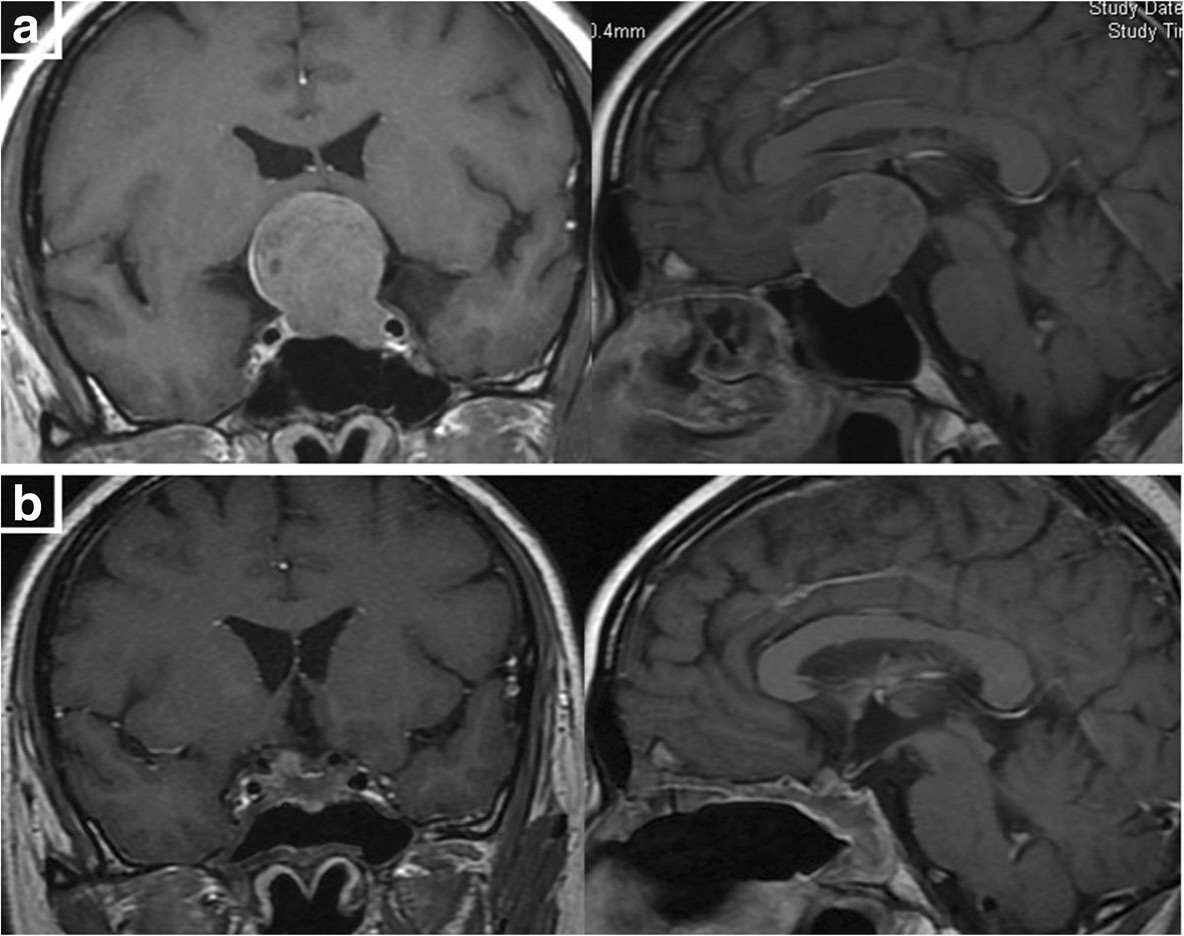
**a** preoperative MRI shows giant pituitary adenoma, growing expansively. **b** postoperative MRI shows tumor was totally removed

In this study, 15 cases in Knosp 1 or 2 growing laterally without cavernous sinus invasion were easier to reach total removal (80%). In 29 cases in Knosp 3, defect can easily be found in medial wall of cavernous sinus under endoscopy. Tumor invading into the cavernous sinus can be removed under direct vision or open the medial wall of cavernous sinus under Doppler monitoring (total removal in 17 cases). In 28 cases in Knosp 4, tumor had involved cavernous sinus and internal carotid artery (ICA), even the lateral wall of cavernous sinus, and cannot be totally removed. Among these cases, 19 soft tumors can be aspirated carefully around ICA to reach near total removal (Figs. 1 and 2) while we withdrew the continued resection in 9 tough tumors. Most of invasive macroadenomas were difficult to be totally removed. However, 29 cases of macroadenomas in Hardy stage III-IV grew expansively, and the direct endoscopic vision and the integrity of tumor capsule make them easily to be removed totally even they were tough (Fig. 3). Further analysis shows that the tumors invading laterally (cavernous sinus etc.) are harder to be removed than those invading vertically (suprasellar, sphenoid sinus, clivus etc.) (*P* = 0.003) (Table 2). Univariate analysis of the clinical data showed that total removal rate was related to tumor volume (*P* = 0.006), reoperation (*P* = 0.011), learning curve (*P* = 0.009) and much closer to tumor invasiveness (*P* < 0.001) (Table 3).

**Table 2:** Analysis of total removal related factors

	Number	Total removal	Non-total	*P* value
Tumor size				
HardyI,II	75	63 (84%)	12 (16%)	0.006
Hardy III,IV	103	66 (64%)	37 (36%)	
Endocrine type				
Functional	86	63 (73%)	23 (27%)	0.868
Non	92	66 (72%)	26 (28%)	
Invasiveness				
Invasive	109	61 (56%)	48 (44%)	0.0001
Non	69	68 (99%)	1 (1%)	
Invading direction				
Purely laterally	44	24 (55%)	20 (45%)	0.003
Purely vertically	37	32 (86%)	5 (14%)	
Knosp Grade				
Knosp 1–2	15	12 (80%)	3 (20%)	0.002
Knosp 3–4	57	18 (32%)	39 (68%)

**Table 3:** Logistic regression analyses identifying predictors of total removal in 178 patients with pituitary adenomas

Factor (category)	Univariate analysis			Multivariate analysis		
	OR	*p* Value	95% CI	OR	*p* Value	95% CI
Volume	0.467	0.006	0.306–0.713		NS	
Invasivenss	0.018	<0.001	0.002–0.134	0.029	0.001	0.004–0.224
Reoperation	0.407	0.011	0.204–0.81		NS	
Learning curve	0.401	0.009	0.203–0.792		NS	

Eighty-six cases in this study (48%) are functional pituitary adenomas. Thirteen prolactinomas in this study invaded cavernous sinus, among which five cases got postoperative imaging total removal, but only three cases meet the remission standard. GH-secreting adenoma can severely affect multiple organic functions. We recommend acromegaly patients taking somatostatin analogue firstly to relieve systemic symptoms and reduce the risk of general anesthesia [4]. In this study, 24 acromegaly patients took long-acting somatostatin analogue for 3 to 6 months preoperatively, which caused the shrinkage of tumor and notable reduce of GH and IGF-1 levels while tumor invasiveness showed no change. Among these patients, tumors were relatively tough and capsules were more obvious, which made tumor easier to be removed totally.

Serious postoperative complications were four cases of intracranial infection, one case of intracranial hemorrhage, and one case die of hydrocephalus.

Intracranial hemorrhage is one of the most serious complications of endoscopic endonasal surgery for two reasons: 1) Residual tumor bleeding, if together with sellar diaphragm rupture, can penetrate to the subarachnoid space or ventricles. 2) The excessive collapse of sellar diaphragm may rupture the artery perforators which adhere to the sellar diaphragm and cause acute bleeding. The former bleeds slowly and can be confirmed by postoperative CT scan. The latter may cause massive hemorrhage and progresses quickly, even lead to death intraoperatively, which makes it hard to handle with. One case had acute intracranial bleeding. In this case, tumor with atypical apoplexy was removed smoothly. When the sellar diaphragm collapsed to sellar floor quickly, diaphragm became bulged and cyan in a sudden. We cut open the diaphragm immediately and proved suprasellar arterial hemorrhage, but the bleeding vessel was not under direct view. A modified pterional approach was done in no time with autologous blood transfusion and volume supplement. At last, the broken artery was clipped and confirmed a branch of right anterior cerebral artery attached to the sellar diaphragm. The patient was recovered well and discharged 10 days later. Through this case, we suggest that excessive sellar diaphragm collapse should be prevent, or reduce the pressure by a positive opening the diaphragm and release of cerebrospinal fluid.

Forty-eight patients (27%) had intraoperative cerebrospinal fluid leakage, which is closely related to tumor size (*P* < 0.001) other than texture (*P* = 0.378). One of the reasons may be the sellar diaphragm would be compressed and weakened by large tumors. The proper use of repair technology can ensure the successful repair. Forty-seven cases (98%) in this study repaired successfully at the first time, which means no recurrence at the long-time follow up. One cerebrospinal rhinorrhea occured at the 10th postoperative day, and was cured with another transsphenoidal repair. The possible reason of failure may be that mucosal flap is too small to cover the skull base. For the defect over 5 mm in diameter or multiple defects, we recommend the multilayer repair technology: we use Absorbable Hemostat (Surgicel, USA) for the first layer to protect the arachnoid and the absorbable artificial dura (DuroGen, USA) for the second to set inside dura; autologous fat, fixed by gelatin sponge, is used to fulfill the dura defect on the third layer. The fourth layer is the pedicled nasal septal mucosal flap to cover the skull base and sealed by biological glue (Fibrin Sealant, China). All these multilayers should be propped by tela iodoformum for 2 weeks. And postoperative lumbar continuous drainage is advised within 5 days. All the skull base reconstruction materials are absorbable or autogenous. Non-absorbable material should be avoided. Four postoperative intracranial infections may be caused by early incomplete nasal disinfection, intra- or postoperative cerebrospinal leak. Further disinfecting the nasal cavity and paranasal sinus with Anerdian and hydrogen peroxide repeatedly, repairing the cerebrospinal leakage reasonably can prevent the infection effectively. One elder patient died of the secondary pneumonia caused by postoperative hydrocephalus.

During the follow-up, five patients with residual nonfunctioning adenomas and 12 patients with residual GH-secreting adenomas underwent stereotactic radiosurgery.

## Discussion

Endoscopic endonasal surgery, which has unlimited potential, provides a new choice for pituitary adenomas to neurosurgeons undoubtedly. However, so far, most neurosurgeons’ unfamiliar to the nasal skull base anatomy or the endoscopic techniques limits the development of this surgery. Other limits may be the lack of cooperation with rhinologists, or the postoperative complications such as cerebrospinal rhinorrhea, secondary intracranial infection. Besides, the neurosurgeons, who are used to the traditional microsurgery, believe that there is no difference in exposure and removal of intrasellar tumor between microscopes and endoscopes. And it cannot be denied that, to the same experienced surgeon, duration under microscope is less than endoscope [5]. Therefore, development of this procedure requires neurosurgeons to keep summarizing how to take the advantage and avoid disadvantage.

### Operative experience

To keep the surgical field clear, it is important to minimize the nasal mucosa damage and mucosa bleeding. After the sellar dura was opened, tumor should be removed in the order: intrasellar firstly, then suprasellar, sellar diaphragm next, the bilateral medial wall of cavernous sinus finally. The adhesion of the suprasellar tumor to sellar diaphram can be found in most cases, which requires careful separation. It is recommended that using a small piece of sponge to protect sellar diaphragm while separate the tumor or capsule, especially in corners. The last step is to check the integrity of bilateral medial wall of cavernous sinus.

Micro-Doppler probe was used mainly in the cases with cavernous sinus invasion. Tumors like this always destroy sellar floor, clivus or other bone landmarks. Compression and displacement of ICA cavernous segment are also common. Thus, the stretch caused by scraping intracavernous tumor blindly may cause ICA or its branch rupture, leading massive hemorrhage. Intraoperative Doppler monitoring can recognize and secure ICA and its branch noninvasively. To stop massive cavernous sinus venous hemorrhage, patient’s head should be lift to reduce the pressure of venous sinus, and one suction tube should be put against the bleeding point. Meanwhile the residual tumor should be keep removing with curette or another suction tube.

### Analysis of total removal related factors

**Fig. 4:**
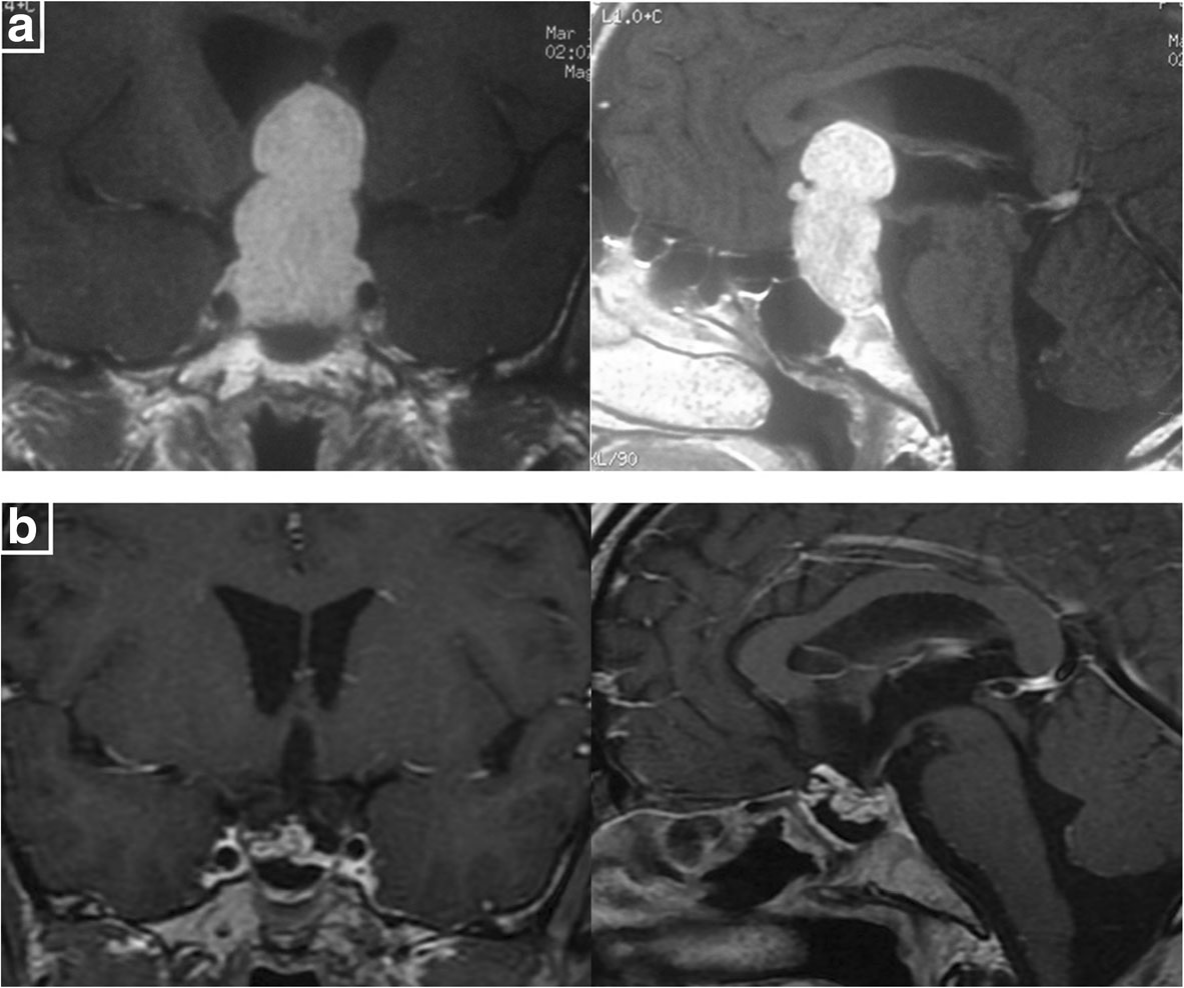
**a** Preoperative MRI shows giant pituitary adenoma, with a dilation of 5.0 cm, compressing foramen of monro. **b** Postoperative MRI shows the total resection of tumor

Once the tumor breaks through the capsule, grows invasively and involves several anatomical spaces (e.g. sellar diaphragm, cavernous sinus, ICA, dorsum sellae, clivus etc.), total removal is seldom achieved. Moreover, the tumors invading laterally are harder to be removed than those invading vertically. One of the reasons may be that the bone structure of sphenoid sinus or clivus can be removed by micro-drill to expand the exposure to reach total removal. It may be another reason that the macroadenomas, which adhere to sellar diaphragm or even break through the diaphragm and compress the third ventricle, can be removed totally by stripping the capsule or open the diaphragm (Fig. 4) while cavernous sinus, involving in lots of vessels and nerves, is hardly to gain enough surgical exposure [6].

### Analysis of endocrinological remission related factors for functional pituitary adenomas

The main surgical goal for functional pituitary adenomas is to achieve the endocrinological remission.

Although the first choice for prolactinomas is drug therapy, we use the operative therapy for those who have surgical indications (resistant to medical therapy, and intolerable adverse medication effects) [7]. Why invasive prolactinomas which got imaging total removal, could not meet the remission endocrinological standard? We speculate that it may be caused by: first, the medial wall of cavernous sinus was infiltrated; second, the tumor capsule was not removed totally. Based on these hypotheses, in later time, we use endoscope to inspect carefully and closely to the tumor cavity and do the biopsy for the fast frozen sections if necessary.

**Fig. 5:**
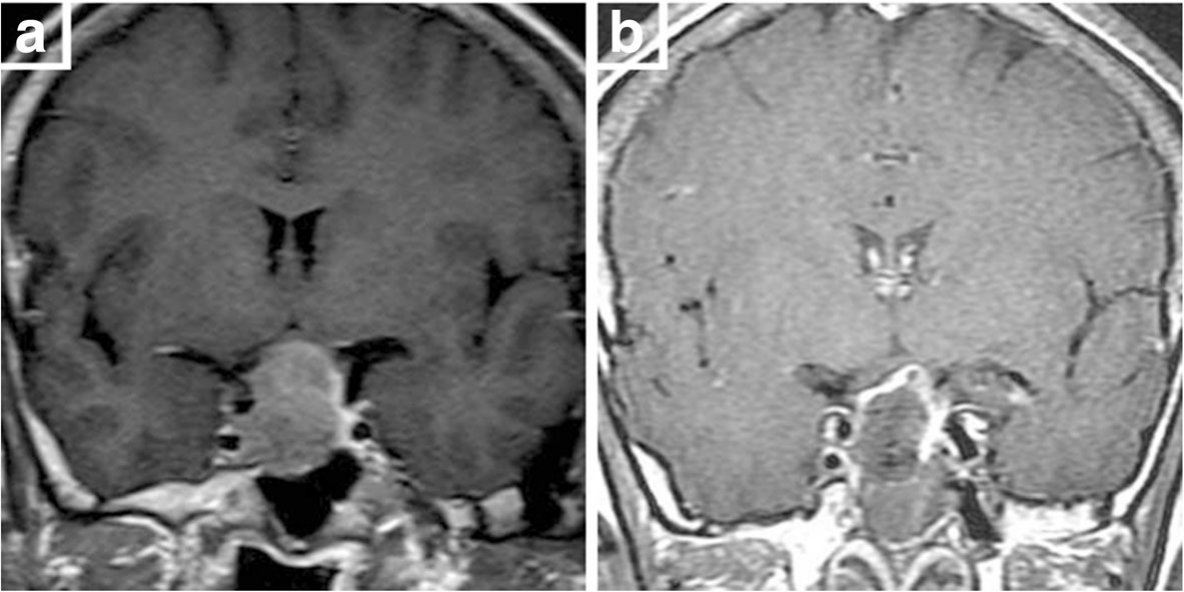
**a** Preoperative MRI shows a GH pituitary macroadenoma of Knosp grade III. **b** Postoperative MRI shows total tumor resection. Endocrinal evaluation on the third postoperative month shows: GH: 0.57 ng/ml, IGF-1: 207 ng/ml (normal range, 94 ~ 284 ng/ml)

To GH-secreting adenomas, just like invasive prolactinomas, the same problem is that although the postoperative imaging shows a total removal of the invasive tumor, endocrinological remission was still not achieved. Among 22 GH-secreting adenomas invading cavernous sinus, 12 got the imaging total removal while only four patients reached biochemical remission (Fig. 5).

Analysis of multiple logistic regression also proves the point above: the endocrinological remission rate is related to tumor invasiveness only (*P* = 0.008) (Table 4).

**Table 4:** Logistic regression analyses identifying predictors of endocrine remission in 86 functional adenomas

Adenoma characteristics	Univariate analysis			Multivariate analysis		
	OR	*p* Value	95% CI	OR	*p* Value	95% CI
Volume	0.310	0.005	0.161–0.597		NS	
Invasiveness	0.137	<0.001	0.051–0.366	0.234	0.008	0.08–0.686

### Learning curve

At the beginning of endoscopic surgery, problems like inadaptation to the endoscopic visualization, the narrow operational space or lack of endoscopic instruments usually showed up. It takes patience and perseverance for neurosurgeons to overcome difficulties, familiarize with and adapt to the endoscopic surgery. Thus, it is recommended to the beginners that operating on those who have a smaller tumor and bigger nasal cavity at the very start. Removal of partial middle turbinate to gain more operational space is also a good choice. Of course, with the company of an experienced ENT doctor, the nasal part of the operation can be done more quickly and neurosurgeons can be more confident. Our own experience is: after accomplishing 30 to 40 endoscopic surgeries, neurosurgeons are more skilled and good teamwork between operator and his assistant is easy to get. Then some harder surgery like invasive pituitary tumor resection can be tried. Besides, we had gathered extraordinary experience for transsphenoidal surgery under microscope for a long time, which shortened our learning curve extremely.

We separate 178 cases into two stages. The ratio of invasive tumor is obviously increased (*P* = 0.009). This is because with the gathering of experience, we realize the specific advantage of invasive pituitary adenoma resection under endoscope and choose more invasive tumors or macroadenomas in the latter stage intentionally, which makes the total removal rate drop from 81 to 64% (*P* = 0.009) unexpectedly. It’s comforting that the remission rate in invasive functional adenomas rises apparently (from 11 to 29%). Besides, because of a thorough disinfection of surgical field, the proficient skill and the improvement of reconstruction of skull base, severe complications (including intracranial infection, intracranial hemorrhage, and postoperative cerebrospinal rhinorrhea) never occurred in the latter stage (Table 5). Compared with other reports [8–10], our imaging total removal rate is similar (72.5%), while endocrinological remission rate of functional pituitary adenoma is relatively lower (44.2%). This may be caused by the very strict endocrinological remission standard and the much higher ratio of invasive adenomas (60.7%).

**Table 5:** Learning curve

	First stage	Second stage	*P* value
Number of cases	89	89	1.000
Total removal	72 (81%)	57 (64%)	0.009
Invasive adenomas	45 (51%)	63 (71%)	0.009
Endocrine remission rate of invasive functional adenomas	2/19 (11%)	10/35 (29%)	0.178
Severe complications*	5	1	0.211

Severe complications*: intracranial infection, intracranial hemorrhage, CSF rhinorrhea and hydrocephalus which required a reoperation

## Conclusions

In conclusion, this study demonstrates that endoscopic endonasal surgery for pituitary adenomas has a high imaging total removal rate (72.5%) while the endocrinological remission rate of functional pituitary adenoma is not that satisfied (44.2%), which means long-time follow up, drug therapy or stereotactic radiotherapy is necessary. The invasion of pituitary adenoma to cavernous sinus is closely related to the surgical results. Due to operator’s elevation of surgical skills and familiarity to endoscopic surgery, tumor removal rate and endocrinological remission rate is improved gradually, with the decrease of the complication rate and the length of learning curve.

## Abbreviations

ACTH: Adrenocorticotrophic hormone
CT: Computed tomography
FSH: Follicle stimulating hormone
GH: Growth Hormone
MR: Magnetic resonance
PRL: Prolactin
TSH: Thyrotropic-stimulating hormone

## References

[1] Schloffer H (1907). Erfolgreiche Operationen eines Hypophysentumors auf Nasalem Wege. Wien Klin Wochenschr.

[2] Jho HD, Carrau RL, Ko Y, Daly MA (1997). Endoscopic pituitary surgery: an early experience. Surg Neurol.

[3] Yaniv E, Rappaport ZH (1997). Endoscopic transseptal transsphenoidal surgery for pituitary tumors. Neurosurgery.

[4] Shen M, Shou X, Wang Y, Zhang Z, Wu J, Mao Y, Li S, Zhao Y (2010). Effect of presurgical long-acting octreotide treatment in acromegaly patients with invasive pituitary macroadenomas: a prospective randomized study. Endocr J.

[5] Fatemi N, Dusick JR, de Paiva Neto MA, Kelly DF (2008). The endonasal microscopic approach for pituitary adenomas and other parasellar tumors: a 10-year experience. Neurosurgery.

[6] Koutourousiou M, Gardner PA, Fernandez-Miranda JC, Paluzzi A, Wang EW, Snyderman CH (2013). Endoscopic endonasal surgery for giant pituitary adenomas: advantages and limitations. J Neurosurg.

[7] Losa M, Mortini P, Barzaghi R, Gioia L, Giovanelli M (2002). Surgical treatment of prolactin-secreting pituitary adenomas: early results and long-term outcome. J Clin Endocrinol Metab.

[8] Han S, Ding X, Tie X, Liu Y, Xia J, Yan A, Wu A (2013). Endoscopic endonasal trans-sphenoidal approach for pituitary adenomas: is one nostril enough?. Acta Neurochir.

[9] Hofstetter CP, Shin BJ, Mubita L, Huang C, Anand VK, Boockvar JA, Schwartz TH (2011). Endoscopic endonasal transsphenoidal surgery for functional pituitary adenomas. Neurosurg Focus.

[10] Gondim JA, Schops M, de Almeida JP, de Albuquerque LA, Gomes E, Ferraz T, Barroso FA (2010). Endoscopic endonasal transsphenoidal surgery: surgical results of 228 pituitary adenomas treated in a pituitary center. Pituitary.

